# Association between the ratio of serum n-3 to n-6 polyunsaturated fatty acids and acute coronary syndrome in non-obese patients with coronary risk factor: a multicenter cross-sectional study

**DOI:** 10.1186/s12872-020-01445-w

**Published:** 2020-04-06

**Authors:** Yuji Nishizaki, Kazunori Shimada, Shigemasa Tani, Takayuki Ogawa, Jiro Ando, Masao Takahashi, Masato Yamamoto, Tomohiro Shinozaki, Tetsuro Miyazaki, Katsumi Miyauchi, Ken Nagao, Atsushi Hirayama, Michihiro Yoshimura, Issei Komuro, Ryozo Nagai, Hiroyuki Daida

**Affiliations:** 1 Department of Cardiovascular Medicine, Juntendo University Graduate School of Medicine, 2-1-1 Hongo Bunkyo-ku, Tokyo, 113-8421 Japan; 2 Medical Technology Innovation Center, Juntendo University, 2-1-1 Hongo Bunkyo-ku, Tokyo, 113-8421 Japan; 3 Department of Cardiology, Nihon University Hospital, 1-6 Kanda surugadai, Chiyoda-ku, Tokyo, 101-8309 Japan; 4 Divison of Cardiology, Department of Internal Medicine, The Jikei University School of Medicine, 3-25-8, Nishi-Shimbashi Minato-ku, Tokyo, 105-8461 Japan; 5 Department of Cardiovascular Medicine, Graduate School of Medicine, The University of Tokyo, 7-3-1 Hongo Bunkyo-ku, Tokyo, 113-8655 Japan; 6 Division of Cardiovascular Medicine, Department of Medicine, Jichi Medical University School of Medicine, 3311-1 Yakushiji Shimotsuke-shi, Tochigi-ken, 329-0498 Japan; 7 Department of Internal Medicine, Tokyo Takanawa Hospital, 3-10-11, Takanawa Minato-ku, Tokyo, 108-8606 Japan; 8 Department of Information and Computer Technology, Faculty of Engineering, Tokyo University of Science, 6-3-1 Niijuku, Katsushika-ku, Tokyo, 125-8585 Japan; 9 Division of Cardiology, Department of Medicine, Nihon University School of Medicine, 30-1 Ohyaguchi Kamichou Itabashi-ku, Tokyo, 173-8610 Japan; 10 Jichi Medical University, 3311-1 Yakushiji Shimotsuke-shi, Tochigi-ken, 329-0498 Japan; 11 Faculty of Health Science, Juntendo University, 2-1-1 Hongo Bunkyo-ku, Tokyo, 113-8421 Japan

**Keywords:** Acute coronary syndrome, Arachidonic acid, Body mass index, Docosahexaenoic acid, Eicosapentaenoic acid, Docosahexaenoic acid/arachidonic acid ratio, Eicosapentaenoic acid/arachidonic acid ratio, Polyunsaturated fatty acids (PUFAs)

## Abstract

**Background:**

Previous studies have reported that being overweight, obese, or underweight is a risk factor for ischemic cardiovascular disease (CVD); however, CVD also occurs in subjects with ideal body mass index (BMI). Recently, the balance of n-3/n-6 polyunsaturated fatty acids (PUFAs) has received attention as a risk marker for CVD but, so far, no study has been conducted that investigates the association between BMI and the balance of n-3/n-6 PUFAs for CVD risk.

**Methods:**

We evaluated the association between n-3/n-6 PUFA ratio and acute coronary syndrome (ACS) in three BMI-based groups (< 25: low BMI, 25–27.5: moderate BMI, and ≥ 27.5: high BMI) that included 1666 patients who visited the cardiovascular medicine departments of five hospitals located in urban areas in Japan.

**Results:**

The prevalence of ACS events was 9.2, 7.3, and 10.3% in the low, moderate, and high BMI groups, respectively. We analyzed the relationship between ACS events and several factors, including docosahexaenoic acid/arachidonic acid (DHA/AA) ratio by multivariate logistic analyses. In the low BMI group, a history of smoking (odds ratio [OR]: 2.47, 95% confidence interval [CI]: 1.40–4.35) and low DHA/AA ratio (OR: 0.30, 95% CI: 0.12–0.74) strongly predicted ACS. These associations were also present in the moderate BMI group but the magnitude of the association was much weaker (ORs are 1.47 [95% CI: 0.54–4.01] for smoking and 0.63 [95% CI: 0.13–3.10] for DHA/AA). In the high BMI group, the association of DHA/AA (OR: 1.98, 95% CI: 0.48–8.24) was reversed and only high HbA1c (OR: 1.46, 95% CI: 1.03–2.08) strongly predicted ACS. The interaction test for OR estimates (two degrees of freedom) showed moderate evidence for reverse DHA/AA ratio–ACS associations among the BMI groups (*P* = 0.091).

**Conclusions:**

DHA/AA ratio may be a useful marker for risk stratification of ACS, especially in non-obese patients.

## Background

The association between body mass index (BMI) and cardiovascular disease (CVD) has recently attracted extensive worldwide attention [1–3]. Although it is easy to understand that obesity adversely affects prognosis, the association between BMI and prognosis in non-obese people has not been fully clarified, and evidence on this issue is particularly deficient for the Asian population. In 2013, Che et al. reported results from a pooled analysis on the relationship between BMI and CVD-related mortality. The BMI versus the CVD-related mortality plot showed a U-shaped curve, revealing negative effects of BMI in the range of ≥25 to < 17.5 on CVD-related mortality [4].

In Japan, the percentage of the elderly population (aged ≥65 years) reached 25% in 2013; it is expected to exceed 30% in 2025 and reach 39.9% in 2060 [5]. As a country experiencing such demographic changes before other countries, Japan is challenged to further extend the healthy life expectancy of the population. As a super-aging society, Japan is expected to see increases in the prevalence of age-related conditions, such as sarcopenia and frailty. While the accepted ideal BMI in Japan is 22, sarcopenic patients, even with a seemingly ideal BMI value, are known to experience many cardiovascular events [6–8]. Therefore, novel cardiovascular event markers need to be identified for non-obese individuals (BMI ≤25), especially in the Asian population.

N-3 polyunsaturated fatty acids (PUFAs) are known to reduce cardiovascular events [9, 10]. Measurement of the serum levels of n-3 PUFAs has served as one of the risk assessment factors for arteriosclerotic disorders. In patients with ischemic heart disease, these levels are useful biomarkers in clinical practice from the viewpoint of managing residual risks. The recently reported results of a large-scale randomized clinical trial (REDUCE-IT: Reduction of Cardiovascular Events With Icosapent Ethyl–Intervention Trial) corroborate this effect [11, 12], and have directed attention toward n-3 PUFAs. Several previous studies have suggested that the n-3/n-6 PUFAs ratio serves as an excellent marker for acute coronary syndrome (ACS) prediction [13–16]. Indeed, we have previously reported that the eicosapentaenoic acid (EPA)/arachidonic acid (AA) ratio, as well as the docosahexaenoic acid (DHA)/AA ratio are useful ACS prediction markers [17–19].

In the present study, we evaluated the association between n-3/n-6 PUFA ratio and ACS in three BMI-based groups (< 25, 25–27.5, and ≥ 27.5) in a total of 1666 patients who visited the cardiovascular medicine departments of five hospitals located in urban areas in Japan.

## Methods

This study was a multicenter cross-sectional study focused on patients who visited the departments of cardiovascular medicine in five centers (one city hospital and four university hospitals) located in Tokyo, Japan. We enrolled 1733 patients who had undergone evaluation of serum PUFAs levels from January 2004 to May 2011. In this analysis, we selected 1666 patients whose BMI information was available. This study included 149 ACS patients and 1517 non-ACS patients.

Acute myocardial infarction is defined as an increase in MB fraction of creatine kinase or troponin T in patients with symptoms of ischemia and/or typical electrocardiographic change (ST elevation). Unstable angina is defined as angina at rest or accelerated exertional angina combined with typical electrocardiographic change (ST depression) and an increased requirement for anti-ischemic therapy [20].

Patients were excluded if they were undergoing hemodialysis or taking purified EPA. Patients with congestive heart failure, severe liver dysfunction, or other systemic diseases, including connective tissue disease and malignancy, were also excluded. This study was approved by the institutional ethics committee of each hospital, and all patients gave informed consent.

We assessed age, sex, BMI, coronary risk factors, medications, and laboratory data, including serum DHA, EPA, dihomo-gamma-linolenic acid (DGLA), and AA. We also evaluated the following data: total cholesterol, triglyceride, low-density lipoprotein cholesterol (LDL-C), high-density lipoprotein cholesterol (HDL-C), hemoglobin A1c (HbA1c), and estimated glomerular filtration rate (eGFR). The eGFR was calculated based on the Japanese equation as follows: eGFR (ml/min/1.73 m2) = 194 × creatinine-1.094 × age-0.287 (female × 0.739) [21]. Serum DHA, EPA, DGLA, and AA levels were measured at an external laboratory (SRL, Inc., Tokyo, Japan). Blood samples were collected from patients either at an outpatient clinic or during admission.

Patients were classified into three groups based on BMI. We evaluated the relationship between the n-3/n-6 PUFAs ratio (DHA/AA ratio and EPA/AA ratio) and ACS events in three BMI groups as follows: low BMI group (BMI < 25), moderate BMI group (25 ≤ BMI < 27.5), and high BMI group (27.5 ≤ BMI). Within each BMI group, we fitted logistic regression for ACS via Firth’s penalized likelihood to deal with sparse outcomes, including sex, age, smoking history, HbA1c, medications (calcium channel blockers and angiotensin II receptor blockers), LDL-C, and n-3/n-6 PUFAs (DHA/AA ratio or EPA/AA ratio) as predictors. The association between ACS risk and DHA/AA ratio were also depicted using natural cubic spline functions. All analyses were performed with SAS version 9.2 (SAS Institute, Cary, NC).

## Results

Table 1 shows the patients’ baseline characteristics according to BMI. The mean ± SD of BMI were 22.3 ± 1.8, 26.0 ± 0.7, and 30.2 ± 2.7 in the low, moderate, and high BMI groups, respectively. The mean ± SD levels of DHA/AA ratio were 0.95 ± 0.41, 0.96 ± 0.31, and 0.90 ± 0.36 in the low, moderate, and high BMI groups, respectively. EPA/AA ratios were 0.49 ± 0.30, 0.50 ± 0.30, and 0.44 ± 0.29 in the low, moderate, and high BMI group, respectively. The prevalence of ACS events was 9.2% (95/1034), 7.3% (27/369), and 10.3% (27/263) in the low, moderate, and high BMI groups, respectively. There were no large differences between the three groups (*P* = 0.39).

**Table 1:** Patient baseline clinical characteristics according to body mass index

	Low BMI group	Moderate BMI group	High BMI group	*P* value
	(*n* = 1034)	(*n* = 369)	(*n* = 263)	
Age (years)	66.3 ± 10.6	63.2 ± 10.5	59.1 ± 12.0	< 0.01*
Male	77.2%	84.8%	74.5%	< 0.01*
Body mass index (kg/m^2^)	22.3 ± 1.8	26.0 ± 0.7	30.2 ± 2.7	< 0.01*
Hypertension	69.8%	78.0%	83.3%	< 0.01*
Diabetes mellitus	36.9%	40.9%	47.1%	< 0.01*
Dyslipidemia	67.6%	73.2%	83.3%	< 0.01*
Smoking history	43.8%	51.8%	46.0%	0.03*
Family history of ischemic heart disease	19.1%	19.8%	18.3%	0.89
Total cholesterol (mg/dl)	189.4 ± 34.6	191.7 ± 36.9	190.1 ± 38.9	0.66
Triglycerides (mg/dl)	138.3 ± 83.8	164.2 ± 117.4	160.9 ± 87.5	< 0.01*
Low-density lipoprotein cholesterol (mg/dl)	109.3 ± 29.8	113.3 ± 29.9	112.0 ± 33.2	0.13
High-density lipoprotein cholesterol (mg/dl)	53.2 ± 16.9	47.5 ± 15.4	48.4 ± 12.8	< 0.01*
Hemoglobin A1c (%)	6.2 ± 1.0	6.3 ± 1.1	6.4 ± 1.0	0.02*
Estimated glomerular filtration rate (ml/min/1.73 m^2^)	68.7 ± 17.2	67.2 ± 17.9	68.8 ± 18.0	0.44
EPA (μg/dl)	72.9 ± 42.6	76.2 ± 45.4	70.3 ± 43.1	0.23
DHA (μg/dl)	141.2 ± 50.0	147.2 ± 54.3	143.8 ± 59.8	0.16
DGLA (μg/dl)	31.9 ± 11.8	34.0 ± 12.4	36.9 ± 13.1	< 0.01*
AA (μg/dl)	154.6 ± 41.3	159.4 ± 71.8	163.2 ± 40.2	0.02*
EPA/AA	0.49 ± 0.30	0.50 ± 0.30	0.44 ± 0.29	0.04*
DHA/AA	0.95 ± 0.41	0.96 ± 0.31	0.90 ± 0.36	0.11
Statins	51.5%	55.8%	61.2%	0.01*
Antiplatelet agents	65.0%	68.0%	58.9%	0.059
Angiotensin II receptor blocker	34.2%	45.8%	44.1%	< 0.01*
Calcium channel blockers	42.6%	54.5%	47.9%	< 0.01*
Beta blockers	38.4%	40.4%	41.1%	0.64
Hypoglycemic agents	20.5%	23.8%	20.9%	0.39
ACS events	9.2%	7.3%	10.3%	0.39

Values are mean ± standard deviation, or percentage

*BMI* Body mass index, *EPA/AA* eicosapentaenoic acid to arachidonic acid ratio, *DHA/AA* docosahexaenoic acid to arachidonic acid ratio, *DGLA* dihomo-gamma-linolenic acid, *ACS* acute coronary syndrome

^a^indicates significance, *p*-values were calculated by chi-squared tests (for binary variables) or analysis of variance (for continuous variables)

**Fig. 1:**
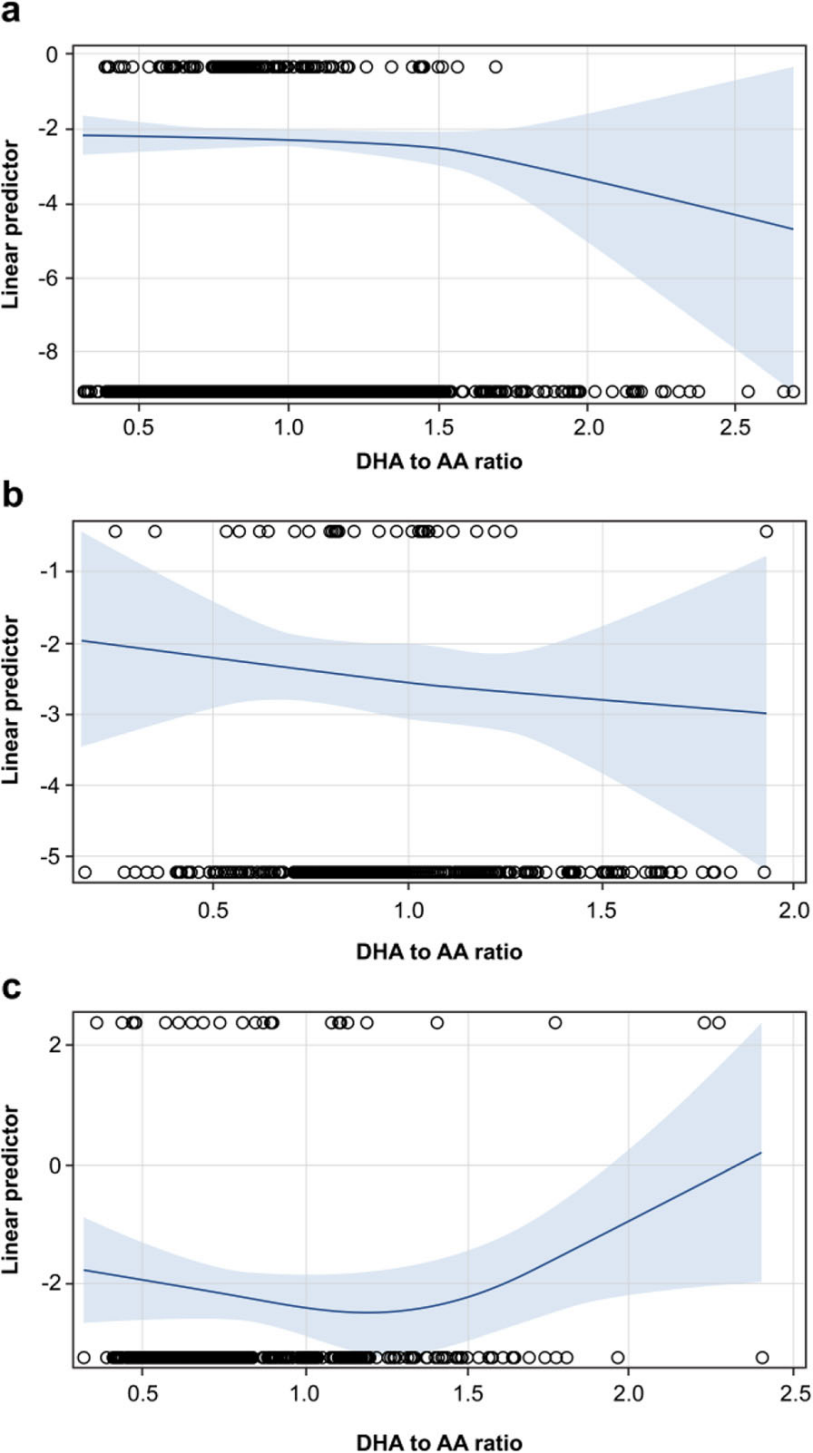
The association between acute coronary syndrome (ACS) and docosahexaenoic acid to arachidonic acid (DHA/AA) ratio. **a** The association between acute coronary syndrome (ACS) and docosahexaenoic acid to arachidonic acid (DHA/AA) ratio by spline curve in low BMI group. **b** The association between ACS and DHA/AA ratio by spline curve in moderate BMI group. **c** The association between ACS and DHA/AA ratio by spline curve in high BMI group

Table 2 shows the estimates of logistic regression model in each BMI group, including DHA/AA ratio as a predictor. In the low BMI group, smoking history (odds ratio [OR]: 2.47, 95% confidence interval [CI]: 1.40–4.35) and low DHA/AA ratio (OR: 0.30, 95% CI: 0.12–0.74) strongly predicted ACS. These associations were also found in the moderate BMI group, but the magnitude of the association was much weaker (ORs are 1.47 [95% CI: 0.54–4.01] for smoking and 0.63 [95% CI: 0.13–3.10] for DHA/AA). In the high BMI group, the association of DHA/AA (OR: 1.98, 95% CI: 0.48–8.24) was reversed and only high HbA1c (OR: 1.46, 95%CI: 1.03–2.08) strongly predicted ACS. The interaction test for OR estimates (two degrees of freedom) showed moderate evidence for reverse DHA/AA ratio–ACS associations among BMI groups (*P* = 0.091). Figures 1a–c show the association between DHA/AA ratio and ACS events by spline curve in each BMI group.

**Table 2:** Odds ratio estimates in multivariable logistic regression for acute coronary syndrome by Firth’s penalized likelihood adjusted for DHA/AA ratio

	BMI group (BMI range)											
	Low BMI (< 25)				Moderate BMI (25–< 27.5)				High BMI (≥27.5)			
	OR	95% CI		*P* value	OR	95% CI		*P* value	OR	95% CI		*P* value
Sex	1.40	0.67	2.94	0.38	0.91	0.24	3.46	0.89	0.88	0.28	2.75	0.83
Age	1.03	1.00	1.06	0.03*	1.03	0.98	1.08	0.20	0.99	0.95	1.03	0.55
Smoking history	2.48	1.41	4.36	0.00*	1.47	0.54	4.01	0.45	0.96	0.36	2.52	0.93
HbA1c	1.08	0.87	1.34	0.51	1.32	0.97	1.80	0.08	1.47	1.03	2.09	0.03*
CCB	1.42	0.83	2.43	0.21	0.77	0.29	2.07	0.60	0.63	0.23	1.78	0.39
ARB	1.06	0.62	1.83	0.83	1.08	0.42	2.77	0.87	0.48	0.17	1.33	0.16
LDL-C	1.00	0.99	1.01	0.48	1.02	1.00	1.03	0.05*	1.00	0.99	1.01	0.85
DHA/AA ratio	0.31	0.13	0.75	0.01*	0.63	0.13	3.10	0.57	1.98	0.48	8.24	0.35

*BMI* body mass index, *SE* standard error, *CI* confidence interval, *HbA1c* hemoglobin A1c, *CCB* calcium channel blocker, *ARB* angiotensin II receptor blocker, *LDL-C* low-density lipoprotein cholesterol, *DHA/AA ratio* docosahexaenoic acid/arachidonic acid ratio

* means *P* < 0.05. All models were fitted in complete cases who did not have missing data: event/n ratios were 61/812 (Low), 19/275 (Moderate), and 20/203 (High)

We also evaluated the association between EPA/AA ratio and ACS events (Table 3). In all BMI groups, we observed relationships similar to those for DHA/AA (OR: 0.41, 95% CI: 0.14–1.17 in low BMI group, OR: 1.12, 95% CI: 0.23–5.44 in moderate BMI group, OR: 2.42, 95% CI: 0.59–9.91 in high BMI group), but the estimates were slightly unstable. The odd ratios for the other variables were essentially the same as in Table 2.

**Table 3:** Odds ratio estimates in multivariable logistic regression for acute coronary syndrome by Firth’s penalized likelihood adjusted for EPA/AA ratio

	BMI group (BMI range)											
	Low BMI (< 25)				Moderate BMI (25–< 27.5)				High BMI (≥27.5)			
	OR	95% CI		*P* value	OR	95% CI		*P* value	OR	95% CI		*P* value
Sex	1.41	0.67	2.97	0.37	0.88	0.23	3.39	0.85	0.87	0.28	2.72	0.80
Age	1.03	1.00	1.06	0.06	1.03	0.98	1.08	0.27	0.99	0.95	1.03	0.53
Smoking history	2.28	1.30	4.00	0.00*	1.46	0.54	3.98	0.46	0.91	0.34	2.42	0.85
HbA1c	1.09	0.88	1.36	0.43	1.34	0.98	1.82	0.07	1.46	1.03	2.08	0.04*
CCB	1.38	0.81	2.36	0.24	0.75	0.28	2.01	0.56	0.59	0.20	1.67	0.32
ARB	1.09	0.64	1.88	0.75	1.07	0.42	2.74	0.89	0.47	0.17	1.31	0.15
LDL-C	1.00	0.99	1.01	0.44	1.02	1.00	1.03	0.05*	1.00	0.98	1.01	0.81
EPA/AA ratio	0.41	0.15	1.18	0.10	1.13	0.23	5.45	0.88	2.42	0.59	9.92	0.22

*BMI* body mass index, *SE* standard error, *CI* confidence interval, *HbA1c* hemoglobin A1c, *CCB* calcium channel blocker, *ARB* angiotensin II receptor blocker, *LDL-C* low-density lipoprotein cholesterol, *EPA/AA ratio* eicosapentaenoic acid/arachidonic acid ratio

* means *P* < 0.05. All models were fitted in complete cases who did not have missing data: event/n ratios were 61/812 (Low), 19/275 (Moderate), and 20/203 (High)

## Discussion

The present study demonstrated that analysis of ACS risk factors in the non-obese (BMI < 25) Japanese population revealed an association between low DHA/AA ratios and ACS.

The prevalence of obesity is increasing in many countries across the world. The World Health Organization (WHO) has estimated that 1 billion adults are overweight and at least 300 million adults are obese [22]. A large number of epidemiological studies have evaluated the possible associations of body weight and BMI with a wide variety of diseases, and have found associations between obesity and various diseases, including type 2 diabetes, hypertension, coronary arterial diseases (CAD), and stroke, as well as several cancers [23]. Previous studies in Asian subjects and the data from cohort studies by the Asia Cohort Consortium (East Asian countries: Japan, China, South Korea, and Singapore; South Asian countries: India and Bangladesh) were used to evaluate the correlation between BMI and CVD-related mortality. A pooled analysis of the data from 1,124,897 subjects in 20 cohorts followed up for a mean duration of 9.7 years revealed that BMI values ≥25 were associated with an elevated risk of CVD death compared with 22.5–24.9 BMI values in East Asians. The risk for CVD death was also higher among people with BMI < 17.5 [4].

The above-mentioned previous studies have reported that being overweight, obese, or underweight is a risk factor for ischemic cardiac diseases; however, some populations are at high risk for cardiovascular events despite having seemingly ideal BMI values, and require caution. Results of a previous study evaluating the relationship between insulin resistance and metabolic abnormalities in non-obese Japanese people have been published and showed that Japanese men with ideal BMI values exhibited muscle insulin resistance when they had hypertension, hyperglycemia, or dyslipidemia [24].

Identifying novel risk factors for ischemic CVD in people with a normal BMI is our own task as Japanese people living in a super-aging society. In the present study, we found that the DHA/AA ratio may be a useful risk factor for ischemic CVD, even in the normal BMI group. To our knowledge, there have been no similar previous reports; hence, this study is novel.

N-3 PUFAs, including DHA and EPA, possess multifaceted functions and reduce arteriosclerosis. Their anti-arteriosclerotic effect is mediated mainly through their triglyceride-reducing action [25]. N-3 PUFAs also have other actions, such as antiplatelet [26, 27], improvement of vascular endothelial function [28–30], anti-inflammatory [31, 32], and blood pressure-lowering actions [33, 34]. Through these multifaceted actions, n-3 PUFAs are considered to reduce arteriosclerosis and thereby prevent cardiovascular events.

In the present study, the observed association between the DHA/AA ratio and ACS events were clearer than that between the EPA/AA ratio and ACS events; however, the cause of this discrepancy is unclear. DHA and EPA are classified as n-3 PUFAs and have similar basic structures, except for the numbers of carbon atoms and double bonds. Compared with EPA, DHA is predominantly found in the phospholipids that form the cell membrane and is thought to play a pivotal role in maintaining the cellular structure. While these structural differences are known between DHA and EPA, future research is necessary to clarify their differences.

Several previous studies have reported the relationship between the DHA/AA ratio or serum DHA level and arteriosclerosis. Nozue et al. measured changes in plaque volume using virtual histology intravascular ultrasound in CVD patients undergoing statin treatment and reported that the DHA/AA ratio was more tightly associated with plaque volume changes than the EPA/AA ratio [35]. Sekikawa et al. reported that the DHA concentration exhibited a stronger correlation with the intima-media thickness (IMT) than the EPA concentration and that this correlation was particularly noticeable in Japanese subjects [36].

This study had several limitations. First, this was a cross-sectional study, and thus causal relationships could not be proven. Second, only patients from Japanese urban areas were included in the study; accordingly, patients in Japanese rural areas or patients in any other country were not included and the generalizability of the results is therefore limited. Third, the number of patients was limited (369 and 263 in the BMI 25–27.5 and ≥ 27.5 groups, respectively). This study focused on BMI, which can be affected by physical activity. The final limitation of this study was that we could not evaluate the information regarding physical activity. A prospective large-scale clinical study is needed to confirm the results of our study.

## Conclusions

This study showed that the DHA/AA ratio may be a useful marker for risk stratification of ACS, especially in non-obese patients (BMI < 25).

## Data Availability

The data sets will not be publicly available because patient consent in each institute do not allow for such publication. The corresponding author will respond to inquiries on data analyses.

## Abbreviations

AA: Arachidonic acid
ACS: Acute coronary syndrome
BMI: Body mass index
CAD: Coronary artery disease
CVD: Cerebro-cardiovascular disease
DGLA: Dihomo-gamma-linolenic acid
DHA: Docosahexaenoic acid
eGFR: Estimated glomerular filtration rate
EPA: Eicosapentaenoic acid
HbA1c: Hemoglobin A1c
HDL-C: High-density lipoprotein cholesterol
IMT: Intima-media thickness
LDL-C: Low-density lipoprotein cholesterol
OR: Odds ratio
PUFAs: Polyunsaturated fatty acids
REDUCE-IT: Reduction of cardiovascular events with Icosapent ethyl–intervention trial
WHO: World Health Organization
95% CI: 95% confidence interval
